# Art Therapy: A Complementary Treatment for Mental Disorders

**DOI:** 10.3389/fpsyg.2021.686005

**Published:** 2021-08-12

**Authors:** Jingxuan Hu, Jinhuan Zhang, Liyu Hu, Haibo Yu, Jinping Xu

**Affiliations:** ^1^College of Creative Design, Shenzhen Technology University, Shenzhen, China; ^2^The Fourth Clinical Medical College of Guangzhou University of Chinese Medicine, Shenzhen, China; ^3^Institute of Biomedical and Health Engineering, Shenzhen Institutes of Advanced Technology, Chinese Academy of Sciences, Shenzhen, China

**Keywords:** painting, art therapy, mental disorders, clinical applications, medical interventions

## Abstract

Art therapy, as a non-pharmacological medical complementary and alternative therapy, has been used as one of medical interventions with good clinical effects on mental disorders. However, systematically reviewed in detail in clinical situations is lacking. Here, we searched on PubMed for art therapy in an attempt to explore its theoretical basis, clinical applications, and future perspectives to summary its global pictures. Since drawings and paintings have been historically recognized as a useful part of therapeutic processes in art therapy, we focused on studies of art therapy which mainly includes painting and drawing as media. As a result, a total of 413 literature were identified. After carefully reading full articles, we found that art therapy has been gradually and successfully used for patients with mental disorders with positive outcomes, mainly reducing suffering from mental symptoms. These disorders mainly include depression disorders and anxiety, cognitive impairment and dementias, Alzheimer’s disease, schizophrenia, and autism. These findings suggest that art therapy can not only be served as an useful therapeutic method to assist patients to open up and share their feelings, views, and experiences, but also as an auxiliary treatment for diagnosing diseases to help medical specialists obtain complementary information different from conventional tests. We humbly believe that art therapy has great potential in clinical applications on mental disorders to be further explored.

## Introduction

Mental disorders constitute a huge social and economic burden for health care systems worldwide (Zschucke et al., 2013; Kenbubpha et al., 2018). In China, the lifetime prevalence of mental disorders was 24.20%, and 1-month prevalence of mental disorders was 14.27% (Xu et al., 2017). The situation is more severely in other countries, especially for developing ones. Given the large numbers of people in need and the humanitarian imperative to reduce suffering, there is an urgent need to implement scalable mental health interventions to address this burden. While pharmacological treatment is the first choice for mental disorders to alleviate the major symptoms, many antipsychotics contribute to poor quality of life and debilitating adverse effects. Therefore, clinicians have turned toward to complementary treatments, such as art therapy in addressing the health needs of patients more than half a century ago.

Art therapy, is defined by the British Association of Art Therapists as: “a form of psychotherapy that uses art media as its primary mode of expression and communication. Clients referred to art therapists are not required to have experience or skills in the arts. The art therapist’s primary concern is not to make an esthetic or diagnostic assessment of the client’s image. The overall goal of its practitioners is to enable clients to change and grow on a personal level through the use of artistic materials in a safe and convenient environment” (British Association of Art Therapists, 2015), whereas as: “an integrative mental health and human services profession that enriches the lives of individuals, families, and communities through active art-making, creative process, applied psychological theory, and human experience within a psycho-therapeutic relationship” (American Art Therapy Association, 2018) according to the American Art Association. It has gradually become a well-known form of spiritual support and complementary therapy (Faller and Schmidt, 2004; Nainis et al., 2006). During the therapy, art therapists can utilize many different art materials as media (i.e., visual art, painting, drawing, music, dance, drama, and writing) (Deshmukh et al., 2018; Chiang et al., 2019). Among them, drawings and paintings have been historically recognized as the most useful part of therapeutic processes within psychiatric and psychological specialties (British Association of Art Therapists, 2015). Moreover, many other art forms gradually fall under the prevue of their own professions (e.g., music therapy, dance/movement therapy, and drama therapy) (Deshmukh et al., 2018). Thus, we excluded these studies and only focused on studies of art therapy which mainly includes painting and drawing as media. Specifically, it focuses on capturing psychodynamic processes by means of “inner pictures,” which become visible by the creative process (Steinbauer et al., 1999). These pictures reflect the psychopathology of different psychiatric disorders and even their corresponding therapeutic process based on specific rules and criterion (Steinbauer and Taucher, 2001). It has been gradually recognized and used as an alternative treatment for therapeutic processes within psychiatric and psychological specialties, as well as medical and neurology-based scientific audiences (Burton, 2009).

The development of art therapy comes partly from the artistic expression of the belief in unspoken things, and partly from the clinical work of art therapists in the medical setting with various groups of patients (Malchiodi, 2013). It is defined as the application of artistic expressions and images to individuals who are physically ill, undergoing invasive medical procedures, such as surgery or chemotherapy for clinical usage (Bar-Sela et al., 2007; Forzoni et al., 2010; Liebmann and Weston, 2015). The American Art Therapy Association describes its main functions as improving cognitive and sensorimotor functions, fostering self-esteem and self-awareness, cultivating emotional resilience, promoting insight, enhancing social skills, reducing and resolving conflicts and distress, and promoting societal and ecological changes (American Art Therapy Association, 2018).

However, despite the above advantages, published systematically review on this topic is lacking. Therefore, this review aims to explore its clinical applications and future perspectives to summary its global pictures, so as to provide more clinical treatment options and research directions for therapists and researchers.

## Publications of Art Therapy

The literatures about “art therapy” published from January 2006 to December 2020 were searched in the PubMed database. The following topics were used: Title/Abstract = “art therapy,” Indexes Timespan = 2006–2020.

**FIGURE 1:**
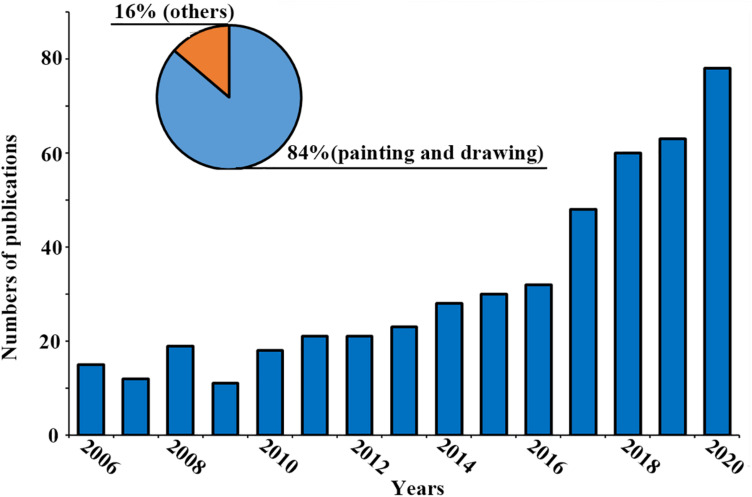
Number of publications about art therapy.

A total of 652 records were found. Then, we manually screened out the literatures that contained the word “art” but was not relevant with the subject of this study, such as state of the art therapy, antiretroviral therapy (ART), and assisted reproductive technology (ART). Finally, 479 records about art therapy were identified. Since we aimed to focus on art therapy included painting and drawing as major media, we screened out literatures deeper, and identified 413 (84%) literatures involved in painting and drawing (Figure 1).

As we can see, the number of literature about art therapy is increasing slowly in the last 15 years, reaching a peak in 2020. This indicates that more effort was made on this topic in recent years (Figure 1).

## Overview of Art Therapy

As defined by the British Association of Art Therapists, art therapy is a form of psychotherapy that uses art media as its primary mode of communication. Based on above literature, several highlights need to be summarized. (1) The main media of art therapy include painting, drawing, music, drama, dance, drama, and writing (Chiang et al., 2019). (2) Main contents of painting and drawing include blind drawing, spiral drawing, drawing moods and self-portraits (Legrand et al., 2017; Abbing et al., 2018; Papangelo et al., 2020). (3) Art therapy is mainly used for cancer, depression and anxiety, autism, dementia and cognitive impairment, as these patients are reluctant to express themselves in words (Attard and Larkin, 2016; Deshmukh et al., 2018; Chiang et al., 2019). It plays an important role in facilitating engagement when direct verbal interaction becomes difficult, and provides a safe and indirect way to connect oneself with others (Papangelo et al., 2020). Moreover, we found that art therapy has been gradually and successfully used for patients with mental disorders with positive outcomes, mainly reducing suffering from mental symptoms. These findings suggest that art therapy can not only be served as an useful therapeutic method to assist patients to open up and share their feelings, views, and experiences, but also as an auxiliary treatment for diagnosing diseases to help medical specialists obtain complementary information different from conventional tests.

## Art Therapy for Mental Disorders

Based on the 413 searched literatures, we further limited them to mental disorders using the following key words, respectively: Depression OR anxiety OR Cognitive impairment OR dementia OR Alzheimer’s disease OR Autism OR Schizophrenia OR mental disorder. As a result, a total of 23 studies (5%) (Table 1) were included and classified after reading the abstract and the full text carefully. These studies include 9 articles on depression and anxiety, 4 articles on cognitive impairment and dementia, 3 articles on Alzheimer’s disease, 3 articles on autism, and 4 articles on schizophrenia. In addition to the English literature, in fact, some Chinese literatures also described the application of art therapy in mental diseases, which were not listed but referred to in the following specific literatures.

**TABLE 1:** Studies of art therapy in mental diseases.

Type of diseases/author and year	Country	Number (painting)	Treatment	Subjects	Main results
**Depression and anxiety**					
Bar-Sela et al., 2007	Israel	19/41	once-weekly, 4w/2w	Cancer patients with depression and anxiety	In the intervention group, the median Hospital Anxiety and Depression Scale score for depression was 9 at the beginning and 7 after the fourth appointment.
Gussak, 2007	Unite States American	48	4-week period, two group sessions per week	Depression	The results reflected a significant decrease in depressive symptoms in those inmates who participated in the program.
Geue et al., 2013	Germany	54	22 sessions	Cancer patients with psychological distress	Anxiety scores decreased in a pre–post comparison.
Crone et al., 2013	United Kingdom	202	10-week intervention	Patients with anxiety, depression, or stress	There was a significant improvement in well-being.
Montag et al., 2014	Germany	58	12 twice-weekly sessions	Acute psychotic episodes with depression	Patients in the ART group showed significant improvement in levels of emotional awareness.
Armstrong and Howatson, 2015	United Kingdom	6 mothers/8 infants	12 consecutive weeks	Postpartum depression	The responses of the questionnaires were more positive after the intervention, and 8 of the 10 mothers showed an improvement in postpartum depression and in the relationship with their children.
Lefèvre et al., 2016	France	28	63 art therapy sessions, 1 h/session	Cancer patients with depression and anxiety	There was a significant reduction in all of the symptoms: pain, anxiety, evil, fatigue, sadness, and depression.
Ciasca et al., 2018	Brazil	31/25	20 weekly art therapy sessions (90 min/session)	Depression	Art therapy as an adjunctive treatment for MDD in the elderly can improve depressive and anxiety symptoms.
Forouzandeh et al., 2020	Iran	55	NA	Anxiety	Nurses should collaborate with medical teams to routinely use non-pharmacological methods such as the painting and the interactive games to alleviate preoperative anxiety in children.
**Cognitive impairment, and dementia**					
Rusted et al., 2016	United Kingdom	45	1 h each week for 40 successive weeks	Dementia	Art therapy is beneficial and appropriate interventions for older people with dementia.
Pike, 2013	Unite States American	91	10-week art therapy	Cognitive impairment	Art therapy treatment was associated with significantly improved cognitive performance.
Heymann et al., 2018	Germany	32	analysis of tree drawings on a digitizing tablet	Mild cognitive impairment (MCI)	MCI group shows a higher recognition rate.
Yu et al., 2021	Singapore	22	weekly 45-min sessions were carried out across 3 months.	MCI	Significant gains in immediate memory and working memory span were observed.
**Alzheimer’s disease**					
Witkoski and Chaves, 2007	Brazil	11	31 months	Alzheimer’s disease	The selection of drawing or modeling showed significant association with severity of cognitive deficit.
Mimica and Kaliniæ, 2011	Croatia	1 case report	not available	Alzheimer’s disease	The art therapy was shown to be an excellent add-on non-pharmacological intervention, beneficial for reducing stress-related behaviors.
Hattori et al., 2011	Japan	39	once weekly for 12 weeks	Alzheimer’s disease	Significant improvement in the quality of life was observed in the art therapy
**Autism**					
Low et al., 2009	New Zealand	27	four 0.5 to 1 h sessions that were approximately 1 week apart	Autism	There is an important relationship between generativity and imagination.
Ten and Muller, 2018	Canada	22	1.5-h session	Autism	There are changes in the type of cognitive processes involved in imagination and children with autism employ a unique cognitive strategy in imaginative drawing.
Jalambadani, 2020	Iran	48	12 sessions	Autism	Autistic children showed more adaptive behaviors and emotions.
**Schizophrenia**					
Richardson et al., 2009	United Kingdom	43	12 weekly sessions of one and a half hours	Schizophrenia	Art therapy produced a statistically significant positive effect on negative symptoms.
Teglbjaerg, 2011	Denmark	5	2 h a week	Schizophrenia	The positive effect of art therapy is mainly due to a strengthening of the Schizophrenia’ minimal sense of self.
Mannheim et al., 2013	Serbian	2	once a week, two months	Schizophrenia	The patient’s drawings show clinical improvement from the reduction of depressive themes and an increase in the frequency of human figure drawings and self-confidence.
Tong et al., 2020	China	104	90 min for a total of 30 times in 15 weeks	Schizophrenia	Group art therapy can improve self-efficacy and social function, reducing social and life function problems, and promote the recovery of individuals diagnosed with schizophrenia.

### Depression Disorders and Anxiety

Depression and anxiety disorders are highly prevalent, affecting individuals, their families and the individual’s role in society (Birgitta et al., 2018). Depression is a disabling and costly condition associated with a significant reduction in quality of life, medical comorbidities and mortality (Demyttenaere et al., 2004; Whiteford et al., 2013; Cuijpers et al., 2014). Anxiety is associated with lower quality of life and negative effects on psychosocial functioning (Cramer et al., 2005). Medication is the most commonly used effective way to relieve symptoms of depression and anxiety. However, nonadherence are crucial shortcomings in using antidepressant to treat depression and anxiety (van Geffen et al., 2007; Nielsen et al., 2019).

In recent years, many studies have shown that art therapy plays a significant role in alleviating depression symptoms and anxiety. Gussak (2007) performed an observational survey about populations in prison of northern Florida and identified that art therapy significantly reduces depressive symptoms. Similarly, a randomized, controlled, and single-blind study about art therapy for depression with the elderly showed that painting as an adjuvant treatment for depression can reduce depressive and anxiety symptoms (Ciasca et al., 2018). In addition, art therapy is also widely used among students, and several studies (Runde, 2008; Zhenhai and Yunhua, 2011) have shown that art therapy also significantly reduces depressive symptoms in students. For example, Wang et al. (2011) conducted group painting therapy on 30 patients with depression for 3 months, and found that painting therapy could promote their social function recovery, improve their social adaptability and quality of life. Another randomized clinical trial also showed that it could decrease mean anxiety scores in the 3–12 year painting group (Forouzandeh et al., 2020).

Studies have shown that distress, including anxiety and depression, is related to poorer health-related quality of life and satisfaction to medical services (Hamer et al., 2009). Painting can be employed to express patients’ anxiety and fear, vent negative emotions by applying projection, thereby significantly improve the mood and reduce symptoms of depression and anxiety of cancer patients. A number of studies (Bar-Sela et al., 2007; Thyme et al., 2009; Lin et al., 2012; Abdulah and Abdulla, 2018) showed that art therapy for cancer patients could enhance the vitality of patients and participation in social activities, significantly reduce depression, anxiety, and reduce stressful feelings. Importantly, even in the follow-up period, art therapy still has a lasting effect on cancer patients (Thyme et al., 2009). Interestingly, art therapy based on famous painting appreciation could also significantly reduce anxiety and depression associated with cancer (Lee et al., 2017). Among cancer patients treated in outpatient health care, art therapy also plays an important role in alleviating their physical symptoms and mental health (Götze et al., 2009). Therefore, art therapy as an auxiliary treatment of cancer is of great value in improving quality of life.

Overall, art painting therapy permits patients to express themselves in a manner acceptable to the inside and outside culture, thereby diminishing depressed and anxiety symptoms.

### Cognitive Impairment, and Dementia

Dementia, a progressive clinical syndrome, is characterized by widespread cognitive impairment in memory, thinking, behavior, emotion and performance, leading to worse daily living (Deshmukh et al., 2018). According to the Alzheimer’s Disease International 2015, there is 46.8 million people suffered from dementia, and numbers almost doubling every 20 years, rising to 131.5 million by 2050. Although art therapy has been used as an alternative treatment for the dementia for long time, the positive effects of painting therapy on cognitive function remain largely unknown. One intervention assigned older adults patients with dementia to a group-based art therapy (including painting) observed significant improvements in the clock drawing test (Pike, 2013), whereas two other randomized controlled trials (Hattori et al., 2011; Rusted et al., 2016) on patients with dementia have failed to obtain significant cognitive improvement in the painting group. Moreover, a cochrane systematic review (Deshmukh et al., 2018) included two clinical studies of art therapy for dementia revealed that there is no sufficient evidence about the efficacy of art therapy for dementia. This may be because patients with severely cognitive impairment, who was unable to accurately remember or assess their own behavior or mental state, might lose the ability to enjoy the benefits of art therapy.

In summary, we should intervene earlier in patients with mild cognitive impairment, an intermediate stage between normal aging and dementia, in order to prevent further transformation into dementia. To date, mild cognitive impairment is drawing much attention to the importance of painting intervening at this stage in order to alter the course of subsequent cognitive decline as soon as possible (Petersen et al., 2014). Recently, a randomized controlled trial (Yu et al., 2021) showed significant relationship between improvement immediate memory/working memory span and increased cortical thickness in right middle frontal gyrus in the painting art group. With the long-term cognitive stimulation and engagement from multiple sessions of painting therapy, it is likely that painting therapy could lead to enhanced cognitive functioning for these patients.

### Alzheimer’s Disease

Alzheimer’s disease (AD) is a sub-type of dementia, which is usually associated with chronic pain. Previous studies suggested that art therapy could be used as a complementary treatment to relief pain for these patients since medication might induce severely side effects. In a multicenter randomized controlled trial, 28 mild AD patients showed significant pain reduction, reduced anxiety, improved quality of life, improved digit span, and inhibitory processes, as well as reduced depression symptoms after 12-week painting (Pongan et al., 2017; Alvarenga et al., 2018). Further study also suggested that individual therapy rather than group therapy could be more optimal since neuroticism can decrease efficacy of painting intervention on pain in patients with mild AD. In addition to release chronic pain, art therapy has been reported to show positive effects on cognitive and psychological symptoms in patients with mild AD. For example, a controlled study revealed significant improvement in the apathy scale and quality of life after 12 weeks of painting treatment mainly including color abstract patterns with pastel crayons or water-based paint (Hattori et al., 2011). Another study also revealed that AD patients showed improvement in facial expression, discourse content and mood after 3-weeks painting intervention (Narme et al., 2012).

### Schizophrenia

Schizophrenia is a complex functional psychotic mental illness that affects about 1% of the population at some point in their life (Kolliakou et al., 2011). Not only do sufferers experience “positive” symptoms such as hallucinations, delusions, but also experience negative symptoms such as varying degrees of anhedonia and asociality, impaired working memory and attention, poverty of speech, and lack of motivation (Andreasen and Olsen, 1982). Many patients with schizophrenia remain symptomatic despite pharmacotherapy, and even attempts to suicide with a rate of 10 to 50% (De Sousa et al., 2020). For these patients, art therapy is highly recommended to process emotional, cognitive and psychotic experiences to release symptoms. Indeed, many forms of art therapy have been successfully used in schizophrenia, whether and how painting may interfere with psychopathology to release symptoms remains largely unknown.

A recent review including 20 studies overall was performed to summary findings, however, concluded that it is not clear whether art therapy leads to clinical improvement in schizophrenia with low (Ruiz et al., 2017). Anyway, many randomized clinical trials reported positive outcomes. For example, Richardson et al. (2007) conducted painting therapy for six months in patients with chronic schizophrenia and found that art therapy had a positive effect on negative symptoms. Teglbjaerg (2011) examined experience of each patient using interviews and written evaluations before and after painting therapy and at a 1-year follow-up and found that group painting therapy in patients with schizophrenia could not only reduce psychotic symptoms, but also boost self-esteem and improve social function.

What’s more, the characteristics of the painting can also be used to judge the health condition in patients with schizophrenia. For example, Hongxia et al. (2013) explored the correlation between psychological health condition and characteristics of House-Tree-Person tests for patients with schizophrenia, and showed that the detail characteristic of the test results can be used to judge the patient’s anxiety, depression, and obsessive-compulsive symptoms.

Most importantly, several other studies showed that drug plus painting therapy significantly enhanced patient compliance and self-cognition than drug therapy alone in patients with schizophrenia (Hongyan and JinJie, 2010; Min, 2010).

### Autism

Autism spectrum disorder (ASD) is a heterogeneous neurodevelopmental syndrome with no unified pathological or neurobiological etiology, which is characterized by difficulties in social interaction, communication problems, and a tendency to engage in repetitive behaviors (Geschwind and Levitt, 2007).

Art therapy is a form of expression that opens the door to communication without verbal interaction. It provides therapists with the opportunity to interact one-on-one with individuals with autism, and make broad connections in a more comfortable and effective way (Babaei et al., 2020). Emery (2004) did a case study about a 6-year-old boy diagnosed with autism and found that art therapy is of great value to the development, growth and communication skills of the boy. Recently, one study (Jalambadani, 2020) using 40 children with ASD participating in painting therapy showed that painting therapy had a significant improvement in the social interactions, adaptive behaviors and emotions. Therefore, encouraging children with ASD to express their experience by using nonverbal expressions is crucial to their development. Evans and Dubowski (2001) believed that creating images on paper could help children express their internal images, thereby enhance their imagination and abstract thinking. Painting can also help autistic children express and vent negative emotions and thereby bring positive emotional experience and promote their self-consciousness (Martin, 2009). According to two studies (Wen and Zhaoming, 2009; Jianhua and Xiaolu, 2013) in China, Art therapy could also improve the language and communication skills, cognitive and behavioral performance of children with ASD.

Moreover, art therapy could be used to investigate the relationship between cognitive processes and imagination in children with ASD. One study (Wen and Zhaoming, 2009; Jianhua and Xiaolu, 2013) suggested that children with ASD apply a unique cognitive strategy in imaginative drawing. Another study (Low et al., 2009) examined the cognitive underpinnings of spontaneous imagination in children with ASD and showed that ASD group lacks imagination, generative ability, planning ability and good consistency in their drawings. In addition, several studies (Leevers and Harris, 1998; Craig and Baron-Cohen, 1999; Craig et al., 2001) have been performed to investigate imagination and creativity of autism via drawing tasks, and showed impairments of autism in imagination and creativity via drawing tasks.

In a word, art therapy plays a significant role in children with ASD, not only as a method of treatment, but also in understanding and investigating patients’ problems.

### Other Applications

In addition to the above mentioned diseases, art therapy has also been adopted in other applications. Dysarthia is a common sequela of cerebral palsy (CP), which directly affects children’s language intelligibility and psycho-social adjustment. Speech therapy does not always help CP children to speak more intelligibly. Interestingly, the art therapy can significantly improve the language intelligibility and their social skills for children with CP (Wilk et al., 2010).

In brief, these studies suggest that art therapy is meaningful and accepted by both patients and therapists. Most often, art therapy could strengthen patient’s emotional expression, self-esteem, and self-awareness. However, our findings are based on relatively small samples and few good-quality qualitative studies, and require cautious interpretation.

## The Application Prospects of Art Therapy

With the development of modern medical technology, life expectancy is also increasing. At the same time, it also brings some side effects and psychological problems during the treatment process, especially for patients with mental illness. Therefore, there is an increasing demand for finding appropriate complementary therapies to improve life quality of patients and psychological health. Art therapy is primarily offered as individual art therapy, in this review, we found that art therapy was most commonly used for depression and anxiety.

Based on the above findings, art therapy, as a non-verbal psychotherapy method, not only serves as an auxiliary tool for diagnosing diseases, which helps medical specialists obtain much information that is difficult to gain from conventional tests, judge the severity and progression of diseases, and understand patients’ psychological state from painting characteristics, but also is an useful therapeutic method, which helps patients open up and share their feelings, views, and experiences. Additionally, the implementation of art therapy is not limited by age, language, diseases or environment, and is easy to be accepted by patients.

Art therapy in hospitals and clinical settings could be very helpful to aid treatment and therapy, and to enhance communications between patients and on-site medical staffs in a non-verbal way. Moreover, art therapy could be more effective when combined with other forms of therapy such as music, dance and other sensory stimuli.

The medical mechanism underlying art therapy using painting as the medium for intervention remains largely unclear in the literature (Salmon, 1993; Broadbent et al., 2004; Guillemin, 2004), and the evidence for effectiveness is insufficient (Mirabella, 2015). Although a number of studies have shown that art therapy could improve the quality of life and mental health of patients, standard and rigorous clinical trials with large samples are still lacking. Moreover, the long-term effect is yet to be assessed due to the lack of follow-up assessment of art therapy.

In some cases, art therapy using painting as the medium may be difficult to be implemented in hospitals, due to medical and health regulations (may be partly due to potential of messes, lack of sink and cleaning space for proper disposal of paints, storage of paints, and toxins of allergens in the paint), insufficient space for the artwork to dry without getting in the way or getting damaged, and negative medical settings and family environments. Nevertheless, these difficulties can be overcome due to great benefits of the art therapy. We thus humbly believe that art therapy has great potential for mental disorders.

In the future, art therapy may be more thoroughly investigated in the following directions. First, more high-quality clinical trials should be carried out to gain more reliable and rigorous evidence. Second, the evaluation methods for the effectiveness of art therapy need to be as diverse as possible. It is necessary for the investigation to include not only subjective scale evaluations, but also objective means such as brain imaging and hematological examinations to be more convincing. Third, it will be helpful to specify the details of the art therapy and patients for objective comparisons, including types of diseases, painting methods, required qualifications of the therapist to perform the art therapy, and the theoretical basis and mechanism of the therapy. This practice should be continuously promoted in both hospitals and communities. Fourth, guidelines about art therapy should be gradually formed on the basis of accumulated evidence. Finally, mechanism of art therapy should be further investigated in a variety of ways, such as at the neurological, cellular, and molecular levels.

## Author Contributions

JH designed the whole study, analyzed the data, and wrote the manuscript. JZ searched for selected the studies. LH participated in the interpretation of data. HY and JX offered good suggestions. All authors read and approved the final manuscript.

## Conflict of Interest

The authors declare that the research was conducted in the absence of any commercial or financial relationships that could be construed as a potential conflict of interest.

## Publisher’s Note

All claims expressed in this article are solely those of the authors and do not necessarily represent those of their affiliated organizations, or those of the publisher, the editors and the reviewers. Any product that may be evaluated in this article, or claim that may be made by its manufacturer, is not guaranteed or endorsed by the publisher.
